# Differentially Expressed MicroRNAs in Postpartum Breast Cancer in Hispanic Women

**DOI:** 10.1371/journal.pone.0124340

**Published:** 2015-04-13

**Authors:** José L. Muñoz-Rodríguez, Lukas Vrba, Bernard W. Futscher, Chengcheng Hu, Ian K. Komenaka, Maria Mercedes Meza-Montenegro, Luis Enrique Gutierrez-Millan, Adrian Daneri-Navarro, Patricia A. Thompson, Maria Elena Martinez

**Affiliations:** 1 The University of Arizona Cancer Center, The University of Arizona, Tucson, AZ, United States of America; 2 Department of Pharmacology and Toxicology, College of Pharmacy, The University of Arizona, Tucson, AZ, United States of America; 3 Department of Epidemiology and Biostatistics, The Mel & Enid Zuckerman College of Public Health, The University of Arizona, Tucson, AZ, United States of America; 4 Department of Surgery, Maricopa Medical Center, Phoenix, AZ, United States of America; 5 Instituto Tecnológico de Sonora, Ciudad Obregón, México; 6 Departamento de Investigaciones Científicas y Tecnológicas, Universidad de Sonora, Hermosillo, México; 7 Departamento de Fisiología, Centro Universitario de Ciencias de la Salud, Universidad de Guadalajara, Guadalajara, México; 8 Department of Cellular and Molecular Medicine, The University of Arizona, Tucson, AZ, United States of America; 9 Department of Family & Preventive Medicine, University of California San Diego, La Jolla, CA, United States of America; Oxford Brookes University, UNITED KINGDOM

## Abstract

The risk of breast cancer transiently increases immediately following pregnancy; peaking between 3-7 years. The biology that underlies this risk window and the effect on the natural history of the disease is unknown. MicroRNAs (miRNAs) are small non-coding RNAs that have been shown to be dysregulated in breast cancer. We conducted miRNA profiling of 56 tumors from a case series of multiparous Hispanic women and assessed the pattern of expression by time since last full-term pregnancy. A data-driven splitting analysis on the pattern of 355 miRNAs separated the case series into two groups: a) an early group representing women diagnosed with breast cancer ≤ 5.2 years postpartum (n = 12), and b) a late group representing women diagnosed with breast cancer ≥ 5.3 years postpartum (n = 44). We identified 15 miRNAs with significant differential expression between the early and late postpartum groups; 60% of these miRNAs are encoded on the X chromosome. Ten miRNAs had a two-fold or higher difference in expression with miR-138, miR-660, miR-31, miR-135b, miR-17, miR-454, and miR-934 overexpressed in the early versus the late group; while miR-892a, miR-199a-5p, and miR-542-5p were underexpressed in the early versus the late postpartum group. The DNA methylation of three out of five tested miRNAs (miR-31, miR-135b, and miR-138) was lower in the early versus late postpartum group, and negatively correlated with miRNA expression. Here we show that miRNAs are differentially expressed and differentially methylated between tumors of the early versus late postpartum, suggesting that potential differences in epigenetic dysfunction may be operative in postpartum breast cancers.

## Introduction

MicroRNAs (miRNAs) are short single-stranded, non-coding RNAs that act as key post-transcriptional regulators of gene expression. MiRNAs exert their function by predominantly binding to the 3’ untranslated region of a target mRNA to stimulate its degradation or inhibit its translation [1]. Based on their targets, certain miRNAs can be categorized into oncogenic miRNAs (oncomirs), while others are considered tumor suppressor miRNAs [2,3]. Tumor suppressor miRNAs act to suppress oncogenes, whereas oncomirs inhibit genes coding for tumor suppressors. Thus, the pattern of expression of tumor suppressor miRNAs and/or the expression of oncogenic miRNAs are thought to be critical regulators of genome integrity and may play an important role in carcinogenesis.

Epigenetic mechanisms, such as DNA methylation, have been shown to regulate miRNA expression [4–7]. Aberrant hypermethylation is associated with silencing of tumor suppressor miRNAs and has been reported in a number of cancers, including breast cancer [8–12]. Recently, a comprehensive analysis of DNA methylation of miRNA genes in breast cancer cell lines and breast cancer tissues found that nearly one third of miRNA promoters were aberrantly methylated in breast cancer cell lines and tissues [9]. Many of these miRNAs have reported tumor suppressor functions. Increased promoter methylation correlated with decreased expression in a subset of the miRNAs queried, highlighting the importance of DNA methylation in miRNA deregulation in breast cancer.

Breast cancer is the second most common cancer diagnosed among women worldwide [13], and the leading cancer diagnosed among women in the United States [14]; including Hispanic women [15]. Several risk factors are associated with breast cancer, including family history, age at first full-term birth, and the number of full-term pregnancies [16]. Results of large prospective studies show that risk of breast cancer also increases in the period immediately following pregnancy [17–22], with the highest transient risk between 3–7 years postpartum [19], and lasting 10 or more years [19–23]. More recently, a distinction has been made between pregnancy-associated breast cancer (occurring during or within one year of pregnancy) from those breast cancers that are diagnosed in the postpartum period (≥ 1 year after pregnancy). This distinction has been made as the postpartum breast cancers, are associated with more aggressive histopathological features, metastasis, and worse outcomes [22]. On average, the postpartum cancers affect younger women and represent a substantial proportion of early onset breast cancers, a phenomenon that disproportionately affects racial ethnic minority populations, such as Hispanic and African American women [24–26].

Understanding that different environmental exposures and pressures, such as age, stress, toxicants, etc., can influence epigenetic state and miRNA expression, we sought to address whether miRNA expression was altered in postpartum breast cancer. Specifically, the goal of this study was to examine miRNA expression and miRNA epigenetic profiles of breast cancers that occur in the transient high-risk postpartum period versus those diagnosed outside of this period. Our results show that in a case series of formalin-fixed, paraffin embedded (FFPE) postpartum associated breast tumor tissue samples, miRNA expression can be separated into two groups based on time since last full-term pregnancy (TSLFTP). Analysis of the miRNA expression identified miRNAs that are differentially expressed between tumors from women diagnosed early after full-term pregnancy (≤ 5.2 years) from those diagnosed later in the postpartum period (≥ 5.3 years). Additionally, we show that the DNA methylation of miRNA genes also differs between the two postpartum groups. Correlation between miRNA expression and DNA methylation of miRNA genes suggests some of these miRNAs are epigenetically regulated. Three miRNAs were identified to have a negative correlation between their expression and DNA methylation; miR-31, miR-135b, and miR-138. A better understanding of miRNA regulation and altered expression in postpartum-associated breast cancer may ultimately yield further insight into the molecular mechanisms of tumorigenesis and new therapeutic strategies against this highly aggressive form of breast cancer.

## Materials and Methods

### Tumor Specimens

Breast tumor tissues, collected and preserved as formalin-fixed paraffin embedded (FFPE) tissue blocks, were obtained from the *Ella* Binational Breast Cancer Study, as previously described [27]. Eligible study participants included women 18 years of age or older, diagnosed with invasive breast cancer at least 12 months after their last full-term pregnancy, and who self-identified as Mexican or Mexican-American. Participants were administered a risk factor questionnaire, and a medical record abstraction was performed to obtain clinical and histopathological information about the specimens. All study participants provided written informed consent, and the Human Studies Committee of the University of Arizona and all other Institutional Review Boards (IRB) at participating sites of the *Ella* Study: 1) University of Sonora Research Bioethics Committee on Investigations; 2) Commission on Ethics, Investigation and Biosafety, University of Guadalajara; 3) Committee on Ethics, Instituto Jaliscience de Cancerologia; 4) National Commission on Scientific Research of the Instituto Nacional del Seguro Social; and 5) Bioethics Institutional Committee for the Instituto Tecnologico de Sonora approved the protocol and consent form used for this study.

Eleven 5 micron (μm) tissue slices were cut using a microtome HM315 Microm (Thermo Fisher Scientific, Wilmington, DE, USA) and placed onto glass microscope slides. A tissue slice was stained with Hematoxylin and Eosin (H&E) as previously described [28]. A pathologist identified and marked the location of the tumor in the tissue slice and determined the percentage of tumor cells present. Only specimens containing greater than 80% tumor cells were analyzed. The subsequent FFPE slides were overlaid onto the H&E slide and the tumor boundaries were marked on the FFPE slides.

### Nucleic Acid Extraction

Tumor tissues were scraped off the microscope slides containing the marked FFPE tumor tissue and placed into a 1.5 mL microfuge tube. Excess and unwanted paraffin was removed by a series of xylene/ethanol washes. The sample was air dried prior to nucleic acid isolation.

Total RNA, including the miRNA, was extracted from five 5 μm FFPE breast tumor tissue slices using the Qiagen miRNeasy FFPE Kit according to the manufacturer’s protocol (Qiagen, Valencia, CA, USA). Genomic DNA was also extracted from five 5 μm FFPE breast tumor tissue slices using the QIAamp DNA FFPE Tissue Kit (Qiagen) according to the manufacturer’s protocol. Quantification of the extracted nucleic acids was performed using the NanoDrop-1000 (Thermo Fisher Scientific, Wilmington, DE, USA).

### Real-time PCR Analysis of miRNA Expression

The miRNAs selected for analysis were previously identified to be expressed in human mammary epithelial cells and fibroblasts [29] that were obtained from the primary cultures of three individuals [30]. The expression of the set of miRNAs contained within our miRNA transcriptome was measured using the EXIQON miRCURY LNA™ Universal RT kit and EXIQON miRCURY LNA™ Universal RT microRNA PCR, Pick & Mix, Ready-to-use Panels (EXIQON, Woburn, MA, USA). The real-time qRT-PCR was carried out as a set of four 96-well plates. Each plate contained 90 unique miRNA primer pairs, three inter-plate calibrators (IPCs; made up of a pre-aliquoted primer and a DNA template) and three reference small RNAs (SNORD38B, SNORD49A, and U6snRNA). The miRNA primers were designed by Exiqon utilizing their locked-nucleic acid (LNA™) technology, which allowed numerous miRNAs to be queried in a single reverse transcriptase reaction in a sensitive and specific manner.

The cDNA was prepared using 120 ng of total RNA according to the manufacture’s protocol (EXIQON), with the exception of the reaction being scaled up three times what is originally specified in the protocol. The cDNA was then diluted (40x) with nuclease-free water (Amresco, Solon, OH, USA). For the real-time PCR, diluted cDNA was combined with PerfeCTA SYBR Green SuperMix, Low ROX (Quanta Biosciences, Gaithersburg, MD, USA) at a 1:1 ratio, and then 10 μL was aliquoted into each well of the set of four real-time PCR plates. Real-time PCR was run on an ABI Prism 7500 Real Time PCR System (Applied Biosystems, Foster City, CA, USA) with a 95°C denaturation for 3 minutes followed by 40 cycles of 95°C for 15 seconds and 60°C for 45 seconds. A dissociation protocol was run after the quantitative RT-PCR, at 95°C for 15 seconds, 60°C for 1 minute and 95°C for 15 seconds.

### Data Analysis

The levels of expression of individual miRNAs were determined as the cycle threshold (Ct) values using the same manual threshold across all the amplicons and samples. The data were imported into R environment [31] and normalized between the plates using the median of IPCs. To adjust for the differences in RNA load and level of degradation between the samples, the data were further normalized between the samples using loess normalization. Ct values were converted into variable increasing with the expression level using formula (45—Ct). Differences in miRNA expression between the sample groups were analyzed using the limma package [32]. Enrichment of X chromosome encoded miRNAs was tested using hypergeometric test as described [33].

### DNA Methylation Analysis by MassARRAY

MassARRAY amplicons 150 to 250 bp in length were targeted to the predicted miRNA transcription start site (TSS) [29] or to miRNA genes. Oligonucleotides used for MassARRAY analysis were designed using EpiDesigner (Sequenom, San Diego, CA, USA) and manufactured by Integrated DNA Technologies (Coralville, IA, USA). Primer sequences are listed in S1 Table.

Genomic DNA (250 ng) from each FFPE breast tumor tissue sample was sodium bisulfite-treated and processed according to the manufacturer’s instructions of the EZ DNA Methylation-Gold™ Kit (Zymo Research Corporation, Irvine, CA, USA). Sodium bisulfite-treated DNA was seeded into a region-specific PCR as described by the manufacturer (Sequenom) and previously reported [34]. Each sodium bisulfite-treated DNA sample was processed as four technical replicates per experiment. The raw data were processed using the EpiTyper software (Sequenom) and further analyzed in the R environment [31]. The data was first filtered by removing data from CpG units having a standard error more than 0.15. Additionally, another filtering was done by discarding samples that had left less than half the CpG units. Differential methylation between the groups was tested using the Wilcoxon test. Spearman correlation coefficient rho between the level of DNA methylation and miRNA expression was calculated using the function cor.test in R.

## Results

### Characteristics of human breast cancer tissue samples

**Fig 1:**
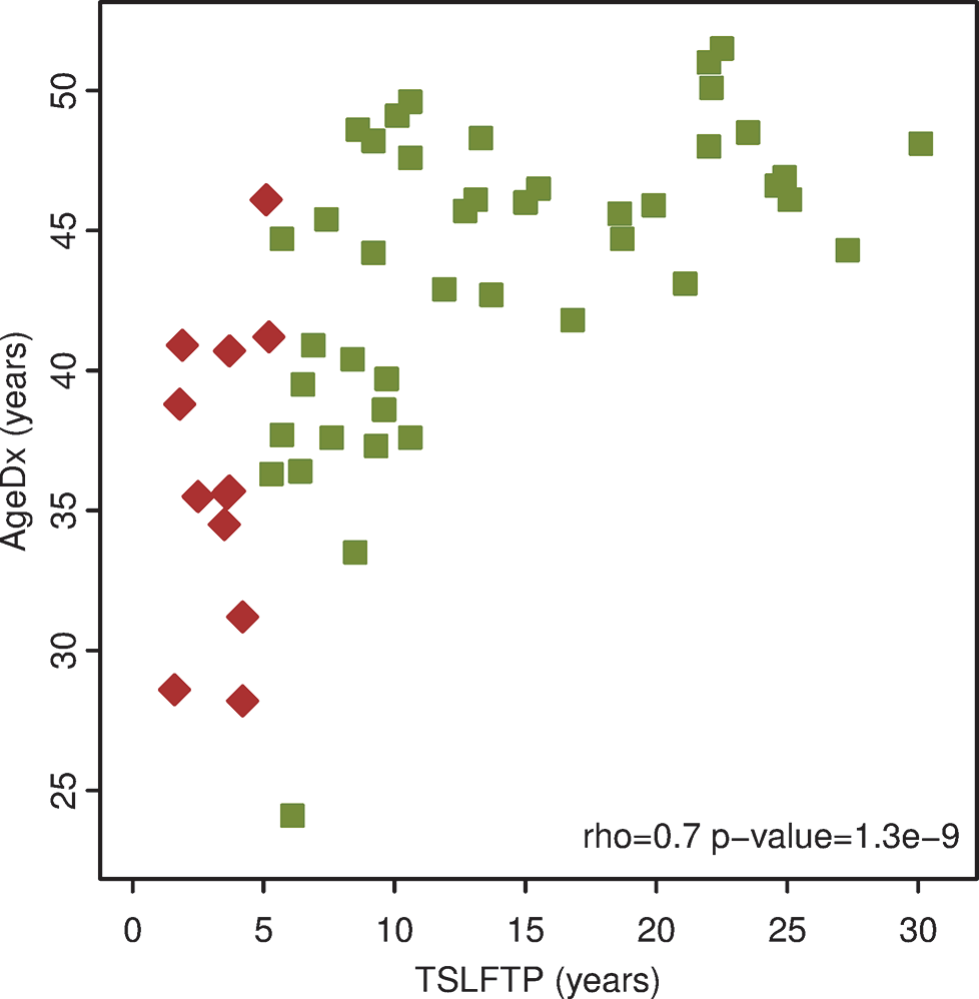
*Ella* sample case series. The graph illustrates distribution of the time since the last full-term pregnancy and the age at diagnosis of the tumor sample case series utilized in the study. The x-axis is the time since last full-term pregnancy (TSLFTP) in years. The y-axis is the age at diagnosis (AgeDx) in years. Red diamonds represent *Ella* FFPE samples ≤ 5.2 years postpartum. Green squares represent *Ella* FFPE samples ≥ 5.3 years postpartum.

Fifty six breast tumor tissues were obtained from the *Ella* Binational Breast Cancer study. The tissues were preserved as FFPE tissue blocks. These samples originated from Hispanic women diagnosed with breast cancer at varying TSLFTP. The range for TSLFTP was 1.6 years to 30.1 years (Fig 1). The study also took into account information on age at diagnosis (AgeDx). The range for AgeDx amongst the samples analyzed was 24.1 years to 51.5 years (Fig 1). Both factors, TSLFTP and AgeDx were found to be highly correlated (rho = 0.7, p-value = 1.3e-9) (Fig 1).

### Natural separation of Ella samples into two groups by miRNA expression

The expression levels of 355 miRNAs across the 56 FFPE breast tumor tissue samples were analyzed utilizing quantitative RT-PCR. We selected this set of miRNAs based on a previous study from our laboratory showing that these miRNAs comprise the human mammary cell miRNA transcriptome [29].

**Fig 2:**
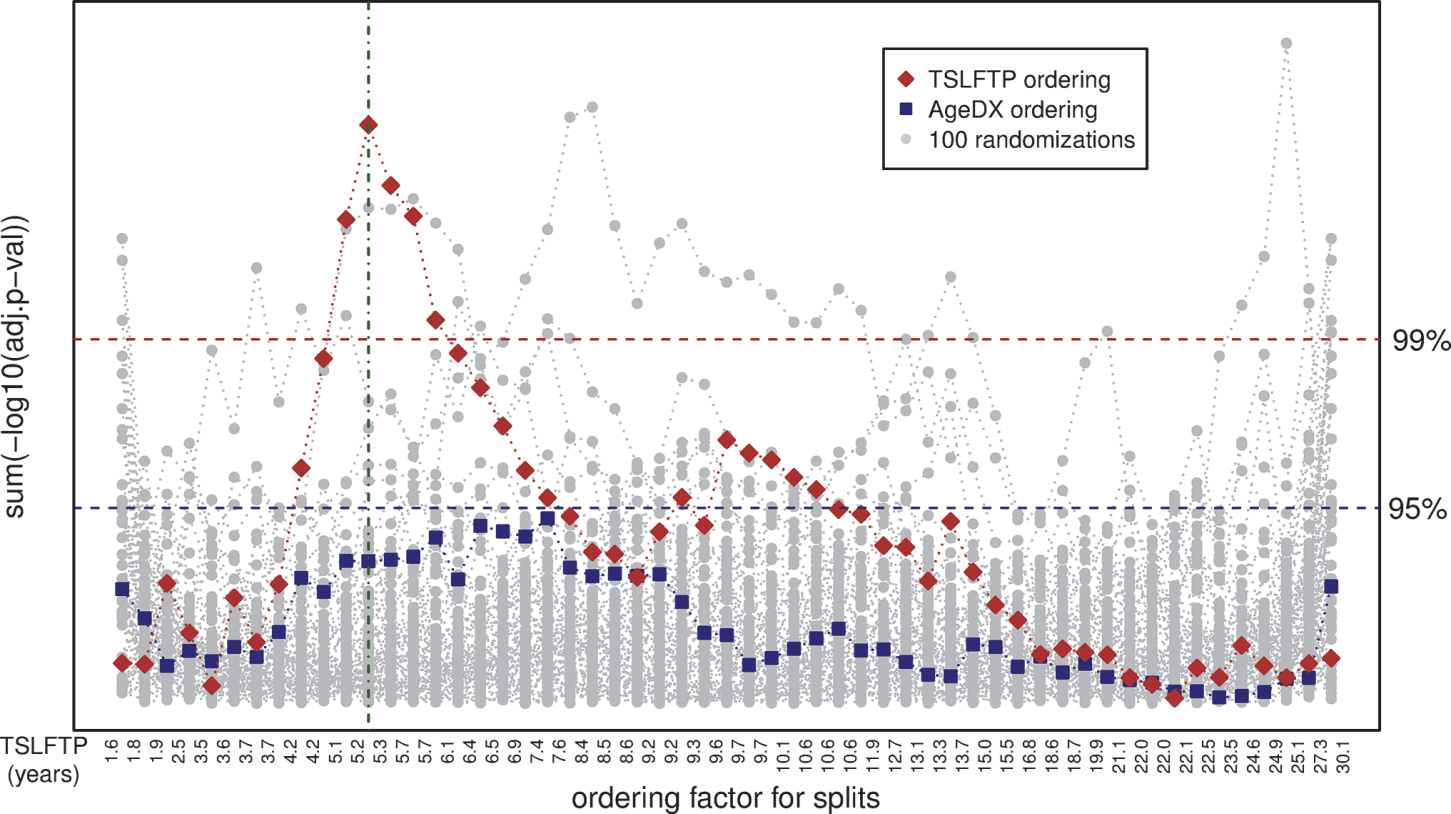
Exhaustive splitting of *Ella* samples. Natural separation of the samples into two groups based on the time since the last full-term pregnancy (TSLFTP). Exhaustive splitting of the *Ella* FFPE case series was based either on TSLFTP, on age at diagnosis (AgeDX), or on randomized data. The x-axis represents individual splits based on each ordering factor. The y-axis represents the level of difference between the groups (sum of the negative log base 10 of the adjusted p-value) for each split. The red diamonds represent the differences after ordering based on TSLFTP. The TSLFTP for individual samples for this ordering is displayed at the bottom. The blue squares are based on ordering by AgeDX. Grey dots represent 100 randomized orderings. Blue and red horizontal dashed lines show the 95th and 99th percentile of the randomized data. Green vertical line indicates the split point with the largest difference between the groups based on TSLFTP.

Given that the exact peak to distinguish early versus late time periods from pregnancy to diagnosis is unknown, we used the miRNA expression data to identify the time-point since last full term pregnancy where miRNA expression differences in the case series are maximal. Since our data clearly show a positive correlation between TSLFTP and AgeDx (Fig 1), we also analyzed the confounding effect of age in the association between TSLFTP and miRNA expression. All the breast cancer samples were ordered based on a) TSLFTP; b) AgeDx; or c) 100 times in a randomized order (Fig 2). The data from tumor samples were then exhaustively split into two groups and the sum of the difference of all miRNAs was calculated to determine the level of difference between the groups. That is, the miRNA expression of the first sample was compared with the miRNA expression of the other 55 samples, then, the miRNA expression of the first two samples was compared to the miRNA expression of the remaining 54 samples, etc. This exhaustive splitting and differential expression calculation revealed that ordering samples based on TSLFTP gave the highest difference in expression between the sample groups, with a maximum between 5.2 and 5.3 years postpartum (Fig 2). No effect was seen when samples were ordered based on AgeDx (Fig 2), indicating that TSLFTP is a factor with much higher impact on miRNA expression than AgeDx. Performing 100 independent randomizations confirmed that the maximum difference of ordering by TSLFTP is significant (fdr < 1%), whereas ordering by AgeDx is not significant (Fig 2). In summary, TSLFTP has a significant effect on miRNA expression level with a natural boundary at 5.25 years postpartum, while the AgeDx falls within the random events. As a result of the exhaustive splitting, the two pregnancy interval groups identified were: a) early group, consisting of 12 samples representing women diagnosed with breast cancer ≤ 5.2 years postpartum and b) late group, consisting of 44 samples representing women diagnosed with breast cancer ≥ 5.3 years postpartum. Of interest, the 5.25 years determined as the natural boundary for miRNA expression is consistent with the cut-point found to empirically determine clinical outcomes in other populations [21,22,35]. Additionally, it is important to note that the pattern of miRNA expression in the patient samples differed by TSLFTP, independent of AgeDx, hormone receptor status, HER2 status, and tumor subtype (S2 Table).

### Differentially expressed miRNAs

**Fig 3:**
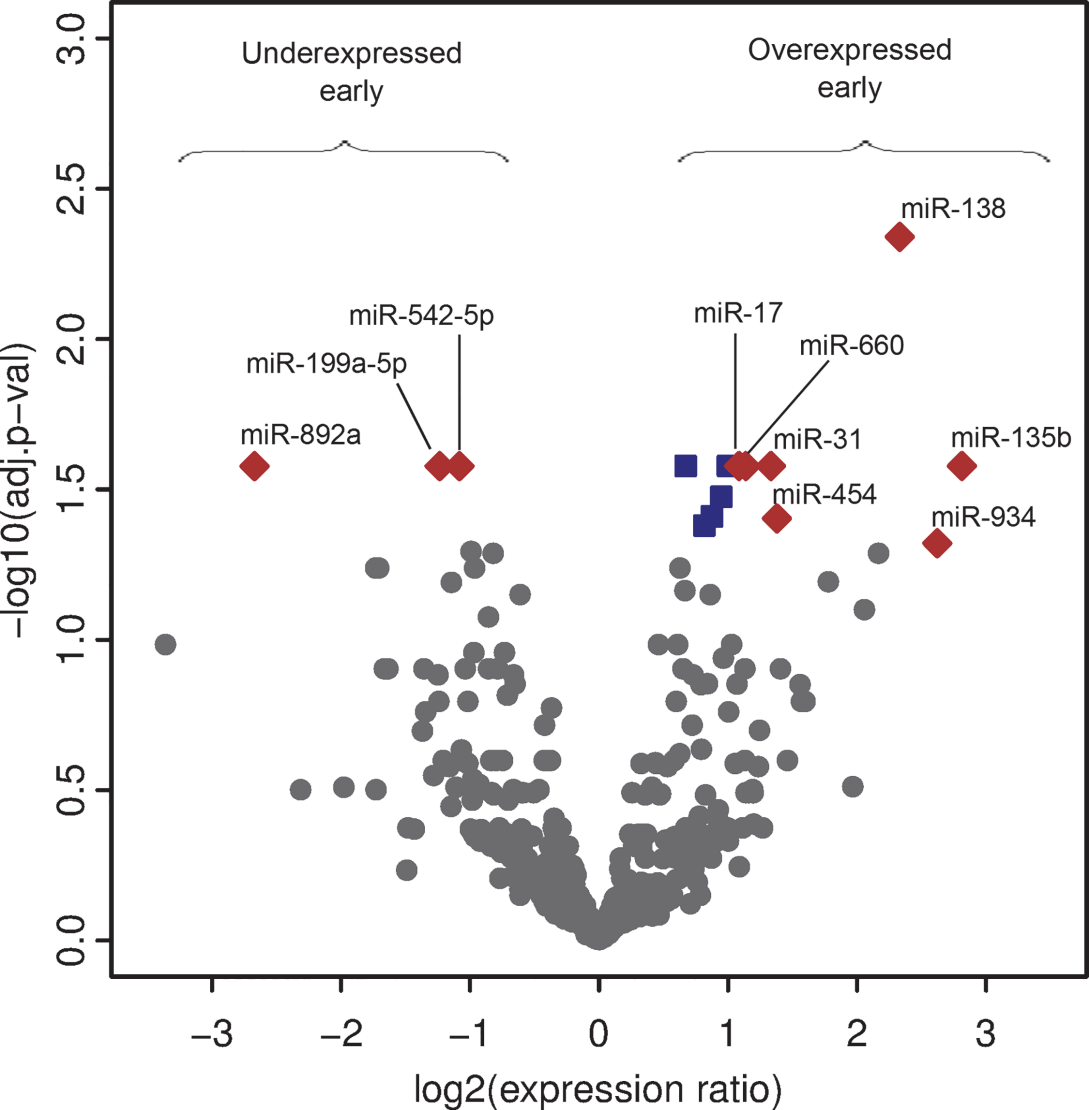
Volcano plot of differentially expressed miRNAs. Comparing miRNAs expressed in *Ella* FFPE case series; early (diagnosed ≤ 5.2 years postpartum) and late (diagnosed ≥ 5.3 years postpartum). The x-axis represents the log base 2 of the miRNA expression ratio. The y-axis represents the negative log base 10 of the adjusted p-value. Red diamonds represent miRNAs meeting stringency cut-off (expression ratio ≥ 2-fold and an adjusted p-value < 0.05). Blue squares represent additional miRNAs with an adjusted p-value < 0.05. Gray dots represent the remaining miRNAs tested.

Next, we assessed the miRNAs that were differentially expressed between the early and late postpartum groups of tumor samples. Out of the 355 miRNAs analyzed, 15 were differentially expressed between the two groups (adjusted p-value < 0.05) (Table 1). Of the 15 differentially expressed miRNAs, 12 were overexpressed in breast cancer from the early compared to the late postpartum group; while 3 were underexpressed in the early compared to the late postpartum group (Table 1, and Fig 3). Seven of the miRNAs overexpressed in breast cancer from the early group reached ≥ 2-fold difference in expression, while all miRNAs underexpressed in breast cancer from the early group also met this criterion (Table 1, and Fig 3)

**Table 1:** MiRNAs differentially expressed between the early and late postpartum breast cancer groups.

miRNA ID	Log_2_ Fold Change	Log_2_ mean Expression	Adjusted p-value	Chromosomes
**miRNAs overexpressed in early group (≤ 5.2 years postpartum)**				
hsa-miR-138	2.331	13.837	0.0046	Chr 3, 16
hsa-miR-660	1.137	15.849	0.0264	Chr X
hsa-miR-31	1.333	18.447	0.0264	Chr 9
hsa-miR-135b	2.813	15.552	0.0264	Chr 1
hsa-miR-500a	0.999	13.462	0.0264	Chr X
hsa-miR-19b	0.672	22.512	0.0264	Chr 13, X
hsa-miR-17	1.085	15.314	0.0264	Chr 13
hsa-miR-362-3p	0.948	5.668	0.0335	Chr X
hsa-miR-106a	0.874	21.138	0.0389	Chr X
hsa-miR-454	1.378	8.447	0.0395	Chr 17
hsa-miR-502-5p	0.816	14.491	0.0417	Chr X
hsa-miR-934	2.621	12.172	0.0477	Chr X
**miRNAs underexpressed in early group (≤ 5.2 years postpartum)**				
hsa-miR-892a	-2.671	8.959	0.0264	Chr X
hsa-miR-199a-5p	-1.236	21.199	0.0264	Chr 1, 19
hsa-miR-542-5p	-1.082	14.069	0.0264	Chr X

Differentially expressed miRNAs. MiRNAs differentially expressed between the two postpartum interval groups identified. Fifty six samples were processed, with early group (≤ 5.2 years postpartum), representing 12 samples, and late group (≥ 5.3 years postpartum), representing 44 samples. MiRNAs identified based on their expression from a quantitative RT-PCR. The listed miRNAs are ordered based on their adjusted p-values. The last column (Chromosomes) indicates on which chromosomes miRNAs are encoded.

We noticed a large portion of differentially expressed miRNAs were encoded on the X chromosome, and tested whether this apparent overrepresentation was statistically significant. We analyzed the expression of 355 mature miRNAs that are potentially encoded by 417 genomic locations, since some miRNAs arise from multiple genes. Of the 355 analyzed miRNAs, 48 (13.5%) are found on the X chromosome. However, 9 of 15 (60%) differentially expressed miRNAs are encoded on the X chromosome (Table 1). This is a highly significant enrichment (p-value = 1.98e-5, hypergeometric test). Most of these X chromosome-encoded, differentially-expressed miRNAs are members of miRNA clusters, which are characterized by a region with one transcription start site, but multiple miRNAs. Four miRNAs overexpressed in the early group (miR-660, miR-500a, miR-362-3p, and miR-502-5p) are part of a cluster mir-532/188/500a/362/501/500b/660/502 encoded on the X chromosome. Two additional miRNAs overexpressed in the early group (miR-106a and miR-19b) are part of an X chromosome encoded cluster mir-106a/18b/20b/19b-2/92a-2/363. Additional analyzed members of this cluster (miR-18b and miR-92a) show a similar fold change towards the same direction as miR-106a and miR-19b, although not significant (S3 Table). MicroRNA 542, a member of the mir-424/503/542/450a-2/450a-1/450b cluster encoded on the X chromosome and some of the additional members encoded by this cluster are co-expressed (S3 Table). MicroRNA 892a is the only analyzed member of another X chromosome encoded miRNA cluster, and finally, miR-934 is encoded on the X chromosome as a single miRNA gene.

In addition to the X chromosome miRNA clusters, two more miRNA gene clusters are represented in the differentially expressed miRNAs. MiRNA 17 and miR-19b are part of the chromosome 13 cluster mir-17/18a/19a/20a/19b-1/92a-1, and again, additional members of this cluster appear co-regulated (S3 Table). Finally, both miRNAs of the mir-301a/454 cluster on chromosome 17 are expressed at higher levels in the early compared to the late postpartum breast cancer group (S3 Table). These observations are consistent with other published data that reports miRNAs found in gene clusters often display a coordinated expression [36]. Taken together, these results suggest that the X chromosome is significantly enriched for miRNAs differentially expressed between early and late postpartum breast cancers.

### DNA Methylation of miRNA regulatory elements

DNA methylation is a known epigenetic regulator of miRNA gene expression [4–7]; therefore, we determined the DNA methylation status of the selected differentially expressed miRNA genes. To detect DNA methylation across miRNA regulatory regions we used the Sequenom MassARRAY platform. We investigated the DNA methylation levels of mir-138-2, mir-135b_TSS, mir-135b_upTSS, mir-31, mir-106a, and mir-542 MassARRAY amplicons (S1 Fig). These miRNA genes were selected for DNA methylation analysis because they displayed differential methylation across breast tumor samples in the microarray data from our previous study [9]. MiRNA 31 was tested across 35 samples, of which 7 represented the early group, and 28 represented the late postpartum group (Fig. B in S1 Fig). The rest of the miRNA MassARRAY amplicons (mir-138-2, mir-135b_TSS, mir-135b_upTSS, mir-106a, and mir-542) were tested on 24 samples, of which 5 represented the early group, and 19 represented the late postpartum group (S1 Fig). Two MassARRAY amplicons were designed for mir-135b to look at two regions within this miRNA gene observed in our laboratory to be differentially methylated across breast tumor samples [9].

**Fig 4:**
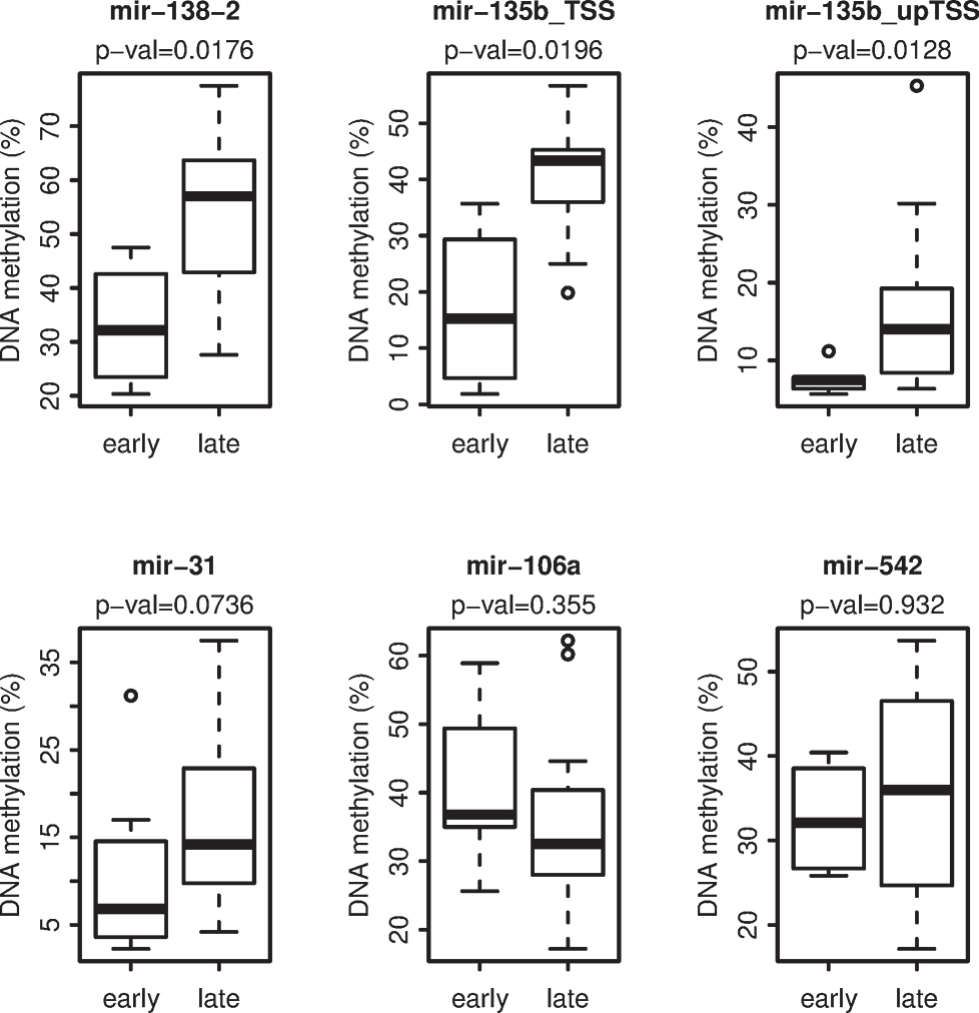
DNA Methylation of selected miRNAs. Box plots illustrating the DNA methylation of the miRNA genes investigated. The names of the miRNA MassARRAY amplicons analyzed by Sequenom are displayed at the top of each plot. The p-value (Wilcoxon test) for each amplicon analyzed is displayed on the second line at the top of the plot. The x-axis is divided into the two interval groups; early, representing *Ella* FFPE samples ≤ 5.2 years postpartum, and late, representing *Ella* FFPE samples ≥ 5.3 years postpartum. The y-axis represents the mean DNA methylation (%) of the amplicon.

The overall DNA methylation levels of the miRNA genes were interpreted as low mean methylation (5%- 30%), moderate mean methylation (31%- 60%) or high mean methylation (> 60%). Low (mir-31, and mir-135b_upTSS) to moderate DNA methylation (mir-138-2, mir-135b_TSS, mir-106a, and mir-542) was seen on the miRNA genes (Fig 4). When comparing the methylation status between the two groups based on TSLFTP, mir-31, mir-135b_TSS, mir-135b_upTSS, and mir-138-2 were less methylated in samples from the early group when compared to the late group (Fig 4), with mir-138-2 and both regions of mir-135b being statistically significant (p < 0.05, Wilcoxon test). MiRNA106a had a higher mean methylation in samples representing the early group when compared to the late group (Fig 4); although not statistically significant. No differences for DNA methylation between the groups were found for mir-542 (Fig 4). Overall, our results suggest that early versus late postpartum breast cancers can be distinguished by differentially expressed miRNAs; some of which also exhibit different levels of DNA methylation.

### Linkage between miRNA expression and DNA methylation

**Fig 5:**
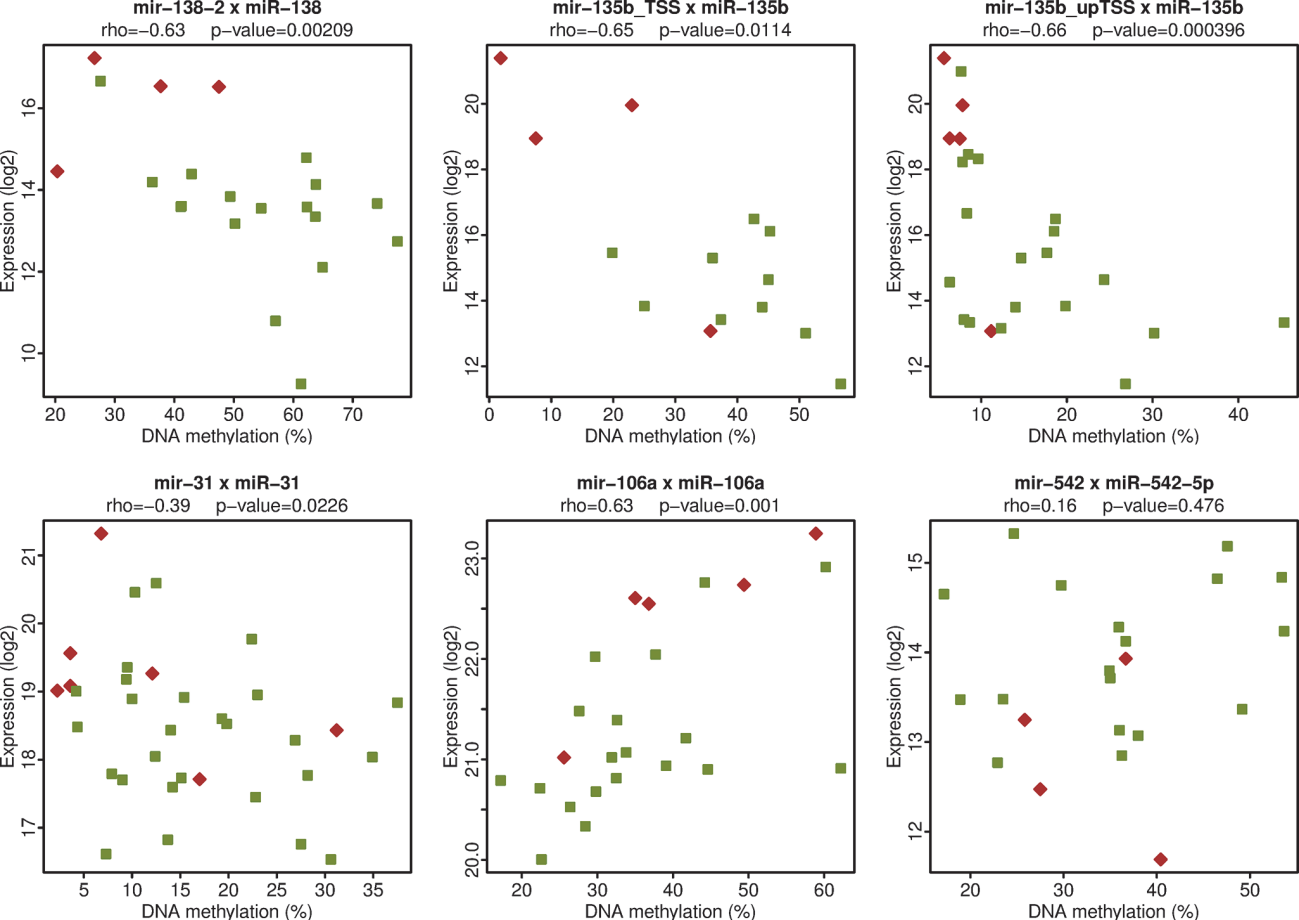
Linkage between miRNA expression and DNA methylation. The x-axis shows the mean DNA methylation (%) of the miRNA Sequenom MassARRAY amplicons. The y-axis shows the miRNA expression levels as determined by quantitative RT-PCR analysis. The names of miRNA genes analyzed by MassARRAY and the names of the respective mature miRNA products detected by quantitative RT-PCR are displayed at the top of each plot. The Spearman correlation coefficient rho and the p-value (two-sided) of the correlation are displayed on the second line at the top of each plot.

We analyzed the relationship between DNA methylation of the miRNA genes and miRNA expression level in our case series. Our data show a correlation between miRNA expression and DNA methylation for some of the miRNAs tested (Fig 5). Specifically, a significant negative correlation between miRNA expression of miR-31, miR-135b and miR-138 and DNA methylation of their genes was shown. MiRNA106a expression had a significant positive correlation with DNA methylation of the miRNA gene (p-value = 0.001). MiRNA 542-5p did not exhibit any correlation (Fig 5). The negative correlation seen between miRNA expression and DNA methylation of miRNA genes for miR-31, miR-135b, and miR-138 indicates that their lower expression in the late postpartum group might be due to increased level of DNA methylation of their genes. These data suggest that differences in miRNA expression between breast cancers occurring in the early versus late postpartum period could be caused by differential DNA methylation and therefore, DNA methylation may contribute to the different general phenotypes of these tumors.

## Discussion

In this study we identified miRNAs that are differentially expressed between breast cancer occurring early and those diagnosed late in the postpartum period in Hispanic women. This was done by analyzing a miRNA transcriptome composed of miRNAs expressed in human mammary cells [29] and identifying specific miRNAs that are differentially expressed within our FFPE case series. Identification of the exact postpartum time point used to distinguish early versus late occurring breast cancers was done utilizing an exhaustive splitting based on the miRNA expression across all of the samples. Importantly, the cut-off of 5.25 years since last full-term pregnancy is consistent with what has been reported in the literature as the peak transient high-risk postpartum period for developing breast cancer [21,22,35]. Furthermore, in a recent published study, Callihan *et al*., showed that women diagnosed within 5 years following a pregnancy had a worse prognosis than those diagnosed during pregnancy or those diagnosed beyond five years postpartum [37], further emphasizing the clinical importance of this transient high-risk period.

To assess if the differentially expressed miRNAs had any association with poorer prognosis, we looked at a dataset from The Cancer Genome Atlas (TCGA). The dataset contained a cohort of 65 women with known time to death or who lived past 10 years post diagnosis. We analyzed the TCGA cohort with respect to the 12 overexpressed and 3 underexpressed miRNAs in the early postpartum group. Consistent with our data demonstrating miR-17 and miR-454 overexpressed in the high risk group (Table 1, and Fig 3), these miRNAs were associated with decreased survival (S2 Fig). The remaining differentially expressed miRNAs identified; however, showed no linkage between miRNA level and survival in the analyzed cohort.

We also evaluated the DNA methylation status of a selected group of differentially expressed miRNAs which allowed us to assess linkage between methylation of miRNA genes and expression of these miRNAs. Three miRNA genes (mir-31, mir-135b [two MassARRAY amplicons], and mir-138-2) were shown to be differentially methylated between the early and late postpartum groups, with the miRNAs having a lower mean methylation for samples in the early postpartum group. It is known that the mature form of miR-138 may originate from two miRNA genes (mir-138-1, and mir-138-2) [38–40]. We only pursued the gene mir-138-2 in our DNA methylation studies, because data mining of previous miRNA methylated DNA immunoprecipitation (MeDIP) microarray data from our lab [9] showed higher methylation variation within the region of mir-138-2 compared to mir-138-1 across human breast samples. The negative correlation between expression and DNA methylation suggest these miRNAs are epigenetically regulated.

We found highly significant enrichment of the X chromosome encoded miRNAs within those differentially expressed between the early and late postpartum groups. These 9 differentially expressed miRNAs are encoded by five independent transcriptional units located on the X chromosome. Three of the transcriptional units were overexpressed and two underexpressed in the early postpartum group. This observation indicates that these miRNAs might be functionally linked to either postpartum group, or their deregulated expression might be an indication of a fundamental deregulation of gene expression of the X chromosome coded genes. One of the X chromosomes in females is epigenetically inactivated throughout the whole life, and the mechanisms that keep one of the X chromosome in active and the other in inactive epigenetic form are tightly regulated. The disruption of this tight regulation in certain tumors may lead to the deregulation of gene expression at the chromosomal level.

X chromosome inactivation (XCI) is a complex epigenetic process responsible for chromosome gene silencing [41,42]. Nevertheless, approximately 15% of X-linked genes are able to escape X inactivation [42–44]. Genes that escape the inactivation are often clustered within large domains between 100 kbp and 7 Mbp [44], and these domains may help explain the high number of differentially expressed, X-linked miRNA genes found in our study (Table 1). The cluster of mir-532/188/500a/362/501/500b/660/502 lies approximately 4 Mbp from the RIBC1 gene, an XCI gene [43]. Likewise, the clusters of mir-106a/18b/20b/19b-2/92a-2/363 and mir-424/503/542/450a-2/450a-1/450b lie within 1.5 Mbp from another XCI gene, GPC4 [43]. The Barr body is frequently absent in breast cancer cells [42,45], and may reflect the duplication of the active X chromosome resulting in the overexpression of X-linked genes [42]. Since we found the differential expression of certain X-linked miRNAs associated with breast cancer in the postpartum period, further investigation of the epigenetic status of the X chromosomes and the gene expression levels of the X chromosome encoded genes might increase our knowledge about the differences between breast tumors from various postpartum periods.

Three miRNAs (miR-31, miR-135b, and miR-138) were identified to have a high fold change and were overexpressed in the early versus late postpartum group. The highly studied miR-31, which was expressed at significantly higher levels in the early postpartum group, has been suggested to act as both a tumor suppressor [46,47] and as an oncogene [47,48]. In a study by Valastyan *et al*., miR-31 expression was found to be inversely associated with metastasis in human breast cancer patients; findings that are inconsistent with reports of more aggressive behavior in the early postpartum group [49]. However, in lung cancer cells, miR-31 has been shown to have oncogenic potential [48]. MiRNA 31 inhibited tumor suppressors, large tumor suppressor, homolog 2 (LATS2) and protein phosphatase 2, regulatory subunit B, alpha (PPP2R2A) [48]. Additionally, our previous study found that miR-31 gene expression correlated with its methylation status that supports an epigenetic regulation of miR-31 [9].

Like miR-31, the levels of miR-135b were significantly higher in the early postpartum group when compared to the late group. Targets of miR-135b include integrin-binding sialoprotein (IBSP) and Sp7 transcription factor (SP7) [50]. MiRNA 135b has previously been reported to be up-regulated in basal human breast cancers and correlated with worse patient survival and metastasis, [51] and separately, to be a member of a set of miRNAs that are predictive of a negative estrogen receptor status [52]. Within our cohort, we observed a negative correlation between miR-135b level and ER or PR status.

MiRNA 138 has been shown to be up-regulated in invasive breast cancer cell lines [53], and may act as a tumor suppressor in breast, endometrial and pancreatic carcinomas via regulation of neutrophil gelatinase-associated lipocalin (NGAL); a gene up-regulated in some cancers [54]. Liu *et al*., investigated the role of miR-138 in epithelial-mesenchymal transition (EMT) and cell migration and invasion in squamous cell carcinoma cell lines [55]. Their study points to the interaction of miR-138 with several target genes involved in EMT; including vimentin (VIM), zinc finger E-box-binding homeobox 2 (ZEB2), and enhancer of zeste homologue 2 (EZH2). They also identified several target genes involved in cell migration and invasion; including Rho-related GTP-binding protein C (RhoC), and Rho-associated, coiled-coil-containing protein kinase 2 (ROCK2) [55]. Identification of differentially expressed miRNAs between the early and the late postpartum groups, such as, miR-31, miR-135b, and miR-138 and their interacting gene partners provide possible ways in which miRNAs may be manipulated in the study of postpartum breast cancers.

Our analysis identified mir-106a having a positive correlation between miRNA expression and DNA methylation of its gene. This miRNA is a member of a cluster, mir-106a/18b/20b/19b-2/92a-2/363 on chromosome X. This cluster is located within a polycomb domain occupied by histone H3 lysine 27 tri-methylation (H3K27me3) [29], a repressive epigenetic mark that negatively correlates with expression. In some cases, there is an antagonism between H3K27me3 and DNA methylation, as was previously reported for the miRNA cluster mir-183/96/182 [29]. The relationship between mir-106a and the polycomb domain may explain why miR-106a is more expressed when more methylated in this study.

## Conclusions

The findings from these studies identify a set of miRNAs that may play a role in postpartum breast cancer in Hispanic women. Fifteen miRNAs were differentially expressed between the early and late postpartum groups with a strong bias towards miRNAs encoded on the X chromosome. Some miRNAs exhibited a differential miRNA gene DNA methylation profile; for example, mir-31, mir-135b, and mir-138 had less mean methylation in the early versus late postpartum group and their DNA methylation negatively correlated with the miRNA gene expression. To the best of our knowledge, this is the first evidence supporting molecular differences between tumors of the early versus late postpartum period. Taken together, these findings also suggest potential differences in epigenetic dysfunction that may be operative in postpartum breast cancers.

Our study is limited by a small sample size, and while our miRNA analysis covered the known mammary miRNA transcriptome, we likely did not detect all differentially expressed miRNAs, such as those not normally expressed in healthy mammary cells, but become activated during breast tumorigenesis. Despite these limitations, our findings provide new information on the miRNAs potentially important in postpartum associated breast cancer.

## Supporting Information

S1 Table DNA Methylation Primers. List of genomic locations of MassARRAY amplicons (hg19 coordinates) and primer sequences for each amplicon obtained from the Sequenom EpiDesigner software.
(PDF)

S2 Table *Ella* case series characteristics. Participant characteristics based on the separation of the samples by time since last full-term pregnancy. The sample case series totals (n = 56), and is restricted to non-neoadjuvant therapy patients. It does include 4 participants who had a tumor sample (biopsy) taken before neoadjuvant therapy was performed.
(PDF)

S3 Table Differentially expressed miRNAs across the sample case series. List of all differentially expressed miRNAs across 56 *Ella* FFPE breast tumor samples processed. List illustrates the miRNA expression data from a quantitative RT-PCR after being analyzed on the R environment using the limma package. A positive log_2_ fold change represents a miRNA overexpressed in the early postpartum group. A negative log_2_ fold change represents a miRNA underexpressed in the early postpartum group. Results are sorted based on the adjusted p-value. The last column (Chromosomes) indicates on which chromosomes miRNAs are encoded.
(XLS)

S1 Fig Sequenom MassARRAY analysis. DNA methylation status across miRNA genes. MiRNAs investigated across 24 *Ella* FFPE breast tumor tissue samples, with the exception of mir-31, which was investigated across 35 samples. Rows represent individual samples (identified with the letter “E”) ordered based on time since last full-term pregnancy, from early to late (numerical values in years on the right-hand side of heatmaps). Columns represent CpG units (labeled at the bottom). Yellow indicates zero to low methylation. Blue indicates high to complete methylation. Dashed boxes indicate no data for that CpG unit, for that specific sample. Filtering of raw methylation data took into account a standard error more than 0.15, and if less than half of the CpG units were left, then that CpG unit was discarded. Dashed horizontal lines divide the early from the late postpartum group. MB231 represents a breast cancer cell line, while, HMEC240LB represents a human mammary epithelial cell line. Both were used as controls for the Sequenom MassARRAY. MB231 data for mir-31 (Fig. B) is missing (dashed boxes) due to a homozygous deletion present in this cell line within the location of the mir-31 gene.
(PDF)

S2 Fig MicroRNAs and survival. Kaplan-Meier plots of differentially expressed miRNAs using TCGA dataset comprised of a cohort of 65 women with known information of time to death or who lived past 10 years post diagnosis. The cohort was split into three groups of 22, 21 and 22 individuals representing low (green line), middle (blue line), and high (red line) level of respective miRNA.
(PDF)
