# Supplementary material for: Differentially Expressed MicroRNAs in Postpartum Breast Cancer in Hispanic Women
Source: PLoS One. 2015 Apr 13;10(4):e0124340. doi: 10.1371/journal.pone.0124340 (PMC4395255; doi:10.1371/journal.pone.0124340)
Supplement: S1 Table — List of genomic locations of MassARRAY amplicons (hg19 coordinates) and primer sequences for each amplicon obtained from the Sequenom EpiDesigner software. (PDF) [file pone.0124340.s001.pdf]

S1 Table. DNA Methylation Primers

Chromosome	Start	End	Amplicon Name	10F Primer Sequence	T7R Primer Sequence
chr9	21,560,136	21,560,334	mir-31	aggaagagagGAGAGGGAGTTTATTTGTAAGAGTTAGAG	cagtaatacgactcactatagggagaaggctAAACCACTCCAAATAAAAAAACACC
chrX	133,307,701	133,307,836	mir-106a	aggaagagagGGTTGTAAGGTTTGTTGTATGAAAA	cagtaatacgactcactatagggagaaggctAAAAAATCCAACCTAAACTACCAAC
chr1	205,425,371	205,425,523	mir-135b_upTSS	aggaagagagTTGAATTTTTGGGAATTGGTATTT	cagtaatacgactcactatagggagaaggctACTACAAAAACCCCCTAAAACCC
chr1	205,418,961	205,419,140	mir-135b_TSS	aggaagagagGGAGGGAGGAGGTTTGTTTT	cagtaatacgactcactatagggagaaggctTAAACCAAAAACTAAAAATCCCAAA
chr16	56,881,571	56,881,768	mir-138-2	aggaagagagTTGTTTTTAAGAGGTTTTAATTAGATTG	cagtaatacgactcactatagggagaaggctCTTTAACTCCCCAACTTCAAAAAA
chrX	133,678,663	133,678,845	mir-542	aggaagagagGGTTTTTTTTGGAGTTAGAAGGAAG	cagtaatacgactcactatagggagaaggctCCCCAAATCAAAATCCCTTAC

List of genomic locations of MassARRAY amplicons (hg19 coordinates) and primer sequences for each amplicon obtained from the Sequenom EpiDesigner software.
