# Supplementary material for: Differentially Expressed MicroRNAs in Postpartum Breast Cancer in Hispanic Women
Source: PLoS One. 2015 Apr 13;10(4):e0124340. doi: 10.1371/journal.pone.0124340 (PMC4395255; doi:10.1371/journal.pone.0124340)
Supplement: S2 Table — Participant characteristics based on the separation of the samples by time since last full-term pregnancy. The sample case series totals (n = 56), and is restricted to non-neoadjuvant therapy patients. It does include 4 participants who had a tumor sample (biopsy) taken before neoadjuvant therapy was performed. (PDF) [file pone.0124340.s002.pdf]

S2 Table. *Ella* case series characteristics.

Characteristic, *n* (%)	Time since last full-term pregnancy ≤ 5.2 y (Early) *n* = 12	≥ 5.3 y (Late) *n* = 44	Total *n* = 56	*P* (*t*-test or Fisher's exact test)
Age at diagnosis, mean ± SD	36.4 ± 5.4	43.6 ± 5.4	42.0 ± 6.1	< 0.001
< 50 y	12 (100.)	41 (93.2)	53 (94.6)	
≥ 50 y	0 (0.00)	3 (6.82)	3 (5.36)	
ER status				0.325
Negative	7 (58.3)	17 (38.6)	24 (42.9)	
Positive	5 (41.7)	27 (61.4)	32 (57.1)	
PR status				0.108
Negative	7 (58.3)	14 (31.8)	21 (37.5)	
Positive	5 (41.7)	30 (68.2)	35 (62.5)	
HER2 status				1.000
Negative	10 (83.3)	34 (77.3)	44 (78.6)	
Positive	2 (16.7)	10 (22.7)	12 (21.4)	
Tumor subtype				0.236
HR+	5 (41.7)	27 (61.4)	32 (57.1)	
HER2+	2 (16.7)	9 (24.3)	11 (19.6)	
TNBC	5 (41.7)	8 (18.2)	13 (23.2)	
Parity				0.523
1–2 children	5 (41.7)	24 (54.6)	29 (51.8)	
3+ children	7 (58.3)	20 (45.5)	27 (48.2)	
Age at first full-term pregnancy				0.840
< 21 y	6 (50.0)	17 (38.6)	23 (41.1)	
21–24 y	2 (16.7)	10 (22.7)	14 (21.5)	
≥ 25 y	4 (33.3)	17 (38.6)	21 (37.5)	

Participant characteristics based on the separation of the samples by time since last full-term pregnancy. The sample case series totals (*n* = 56), and is restricted to non-neoadjuvant therapy patients. It does include 4 participants who had a tumor sample (biopsy) taken before neoadjuvant therapy was performed.
