# Supplementary material for: Differentially Expressed MicroRNAs in Postpartum Breast Cancer in Hispanic Women
Source: PLoS One. 2015 Apr 13;10(4):e0124340. doi: 10.1371/journal.pone.0124340 (PMC4395255; doi:10.1371/journal.pone.0124340)
Supplement: S1 Fig — DNA methylation status across miRNA genes. MiRNAs investigated across 24 Ella FFPE breast tumor tissue samples, with the exception of mir-31, which was investigated across 35 samples. Rows represent individual samples (identified with the letter “E”) ordered based on time since last full-term pregnancy, from early to late (numerical values in years on the right-hand side of heatmaps). Columns represent CpG units (labeled at the bottom). Yellow indicates zero to low methylation. Blue indicates high to complete methylation. Dashed boxes indicate no data for that CpG unit, for that specific sample. Filtering of raw methylation data took into account a standard error more than 0.15, and if less than half of the CpG units were left, then that CpG unit was discarded. Dashed horizontal lines divide the early from the late postpartum group. MB231 represents a breast cancer cell line, while, HMEC240LB represents a human mammary epithelial cell line. Both were used as controls for the Sequenom MassARRAY. MB231 data for mir-31 (Fig. B) is missing (dashed boxes) due to a homozygous deletion present in this cell line within the location of the mir-31 gene. (PDF) [file pone.0124340.s004.pdf]

Figure A:

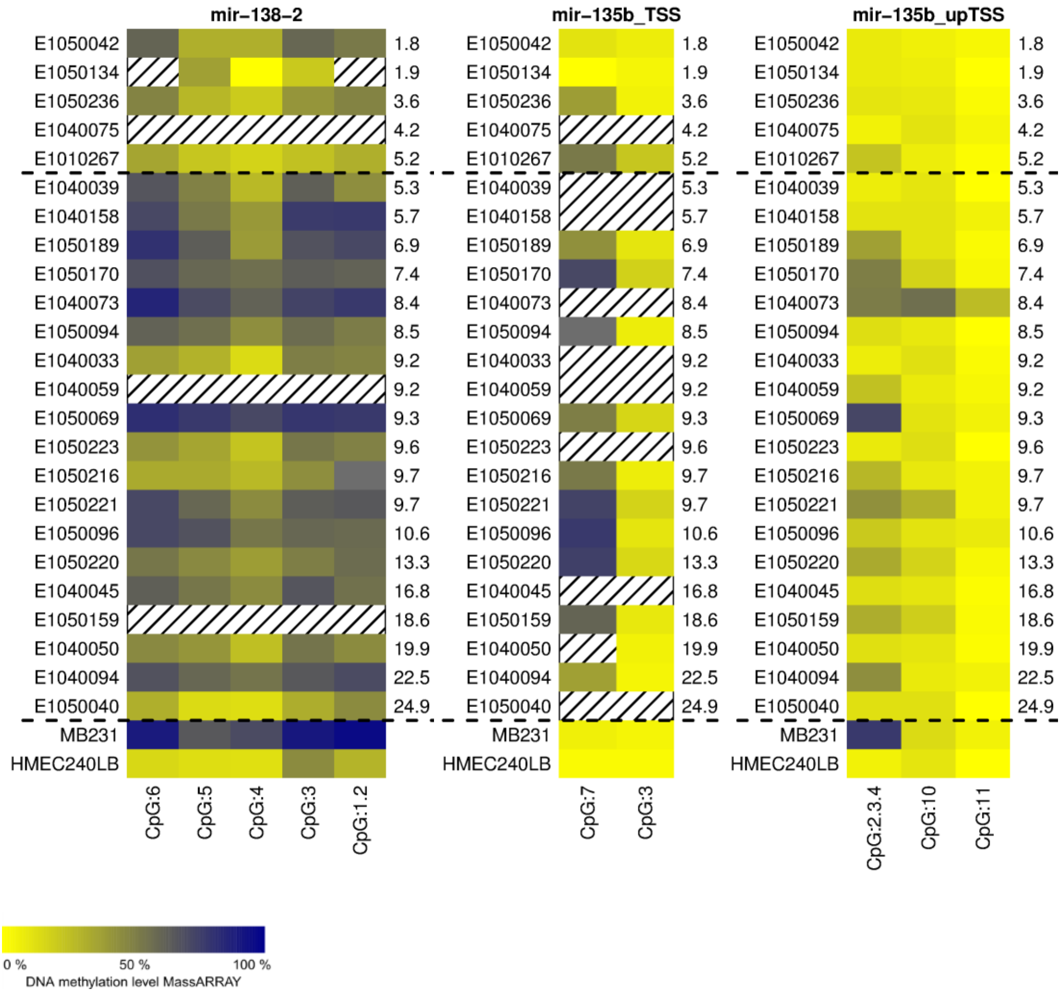
mir−138−2
mir−135b_TSS
mir−135b_upTSS
E1050042 1.8
E1050134 1.9
E1050236 3.6
E1040075 4.2
E1010267 5.2
E1040039 5.3
E1040158 5.7
E1050189 6.9
E1050170 7.4
E1040073 8.4
E1050094 8.5
E1040033 9.2
E1040059 9.2
E1050069 9.3
E1050223 9.6
E1050216 9.7
E1050221 9.7
E1050096 10.6
E1050220 13.3
E1040045 16.8
E1050159 18.6
E1040050 19.9
E1040094 22.5
E1050040 24.9
MB231
HMEC240LB
CpG:6
CpG:5
CpG:4
CpG:3
CpG:1.2
CpG:7
CpG:3
CpG:2.3.4
CpG:10
CpG:11
0 %
50 %
100 %
DNA methylation level MassARRAY

Figure B:

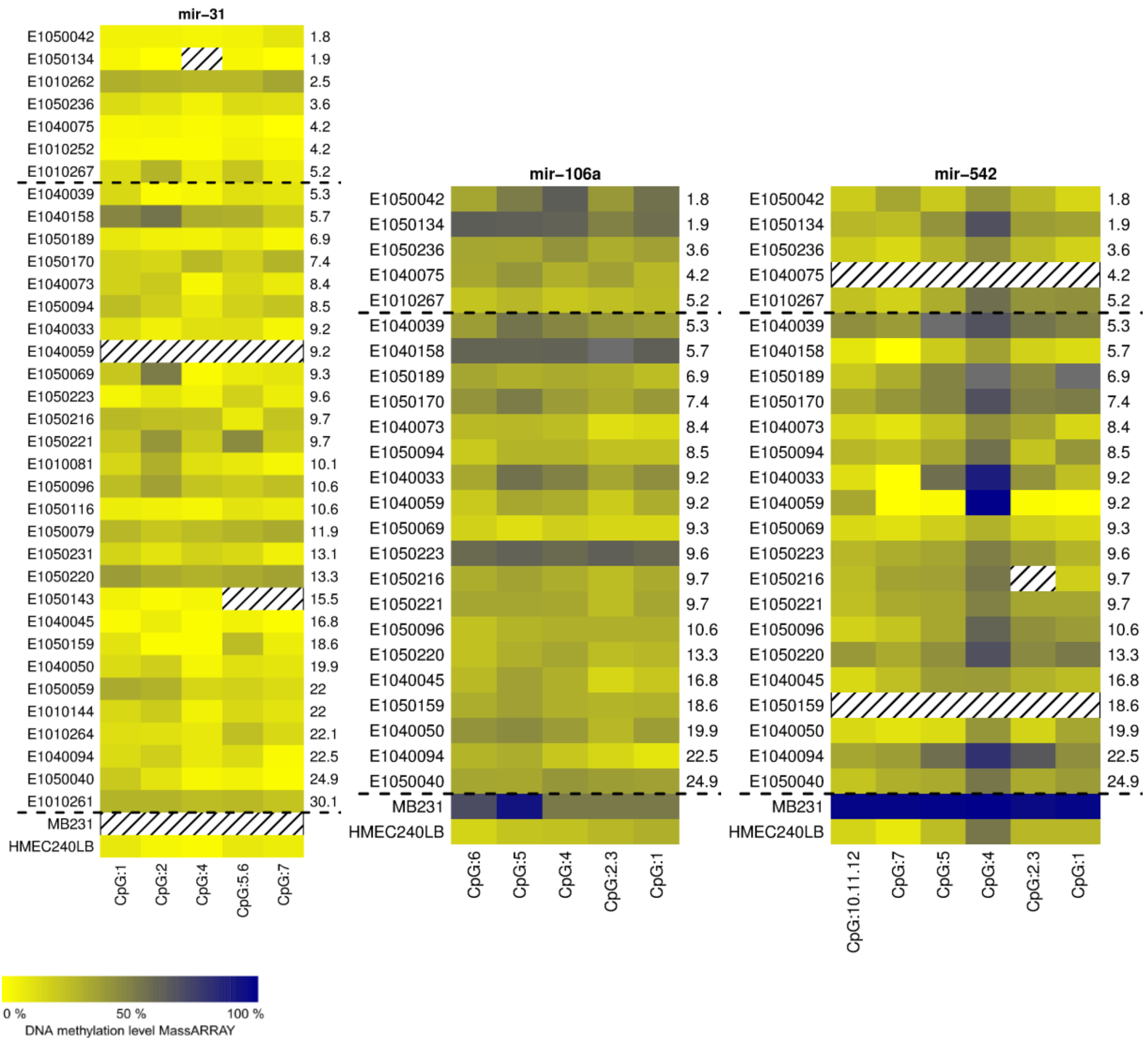

S1 Figure. Sequenom MassARRAY analysis. DNA methylation status across miRNA genes. MiRNAs investigated across 24 *Ella* FFPE breast tumor tissue samples, with the exception of mir-31, which was investigated across 35 samples. Rows represent individual samples (identified with the letter "E"), ordered based on time since last full-term pregnancy, from early to late (numerical values in years on the right-hand side of heatmaps). Columns represent CpG units (labeled at the bottom). Yellow indicates zero to low methylation. Blue indicates high to complete methylation. Dashed boxes indicate no data for that CpG unit, for that specific sample. Filtering of raw methylation data took into account a standard error more than 0.15, and if less than half of the CpG units were left, then that CpG unit was discarded. Dashed horizontal lines divide the early from the late postpartum group. MB231 represents a breast cancer cell line, while, HMEC240LB represents a human mammary epithelial cell line. Both were used as controls for the Sequenom MassARRAY. MB231 data for mir-31 (Figure B) is missing (dashed boxes) due to a homozygous deletion present in this cell line within the location of the mir-31 gene.
