# Supplementary material for: Differentially Expressed MicroRNAs in Postpartum Breast Cancer in Hispanic Women
Source: PLoS One. 2015 Apr 13;10(4):e0124340. doi: 10.1371/journal.pone.0124340 (PMC4395255; doi:10.1371/journal.pone.0124340)
Supplement: S2 Fig — Kaplan-Meier plots of differentially expressed miRNAs using TCGA dataset comprised of a cohort of 65 women with known information of time to death or who lived past 10 years post diagnosis. The cohort was split into three groups of 22, 21 and 22 individuals representing low (green line), middle (blue line), and high (red line) level of respective miRNA. (PDF) [file pone.0124340.s005.pdf]

Figure A:

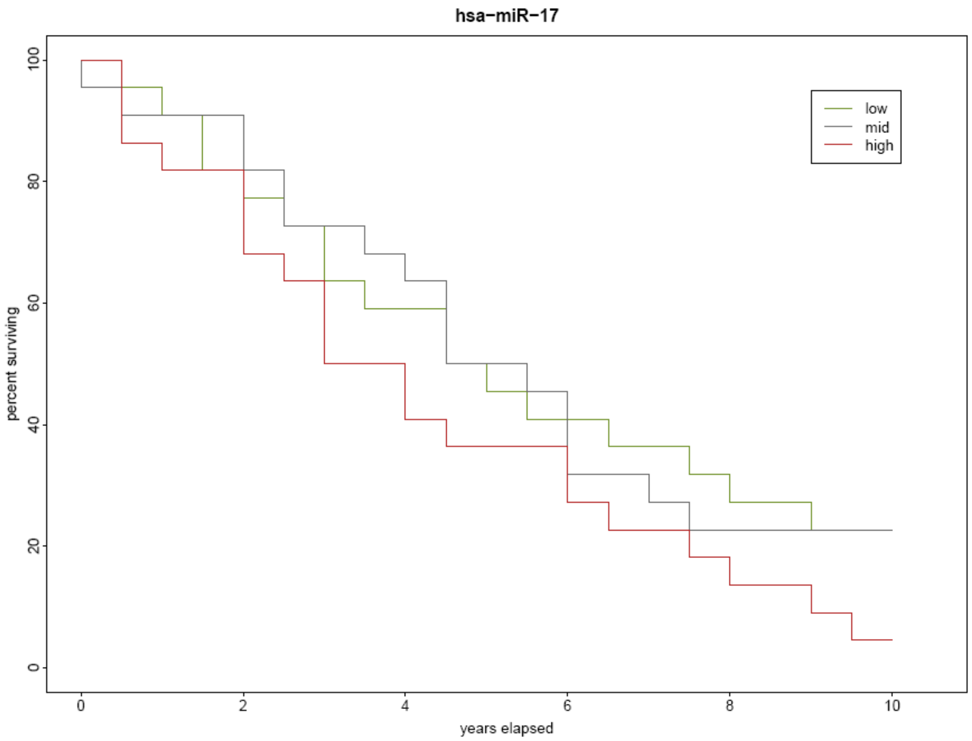

Figure B:

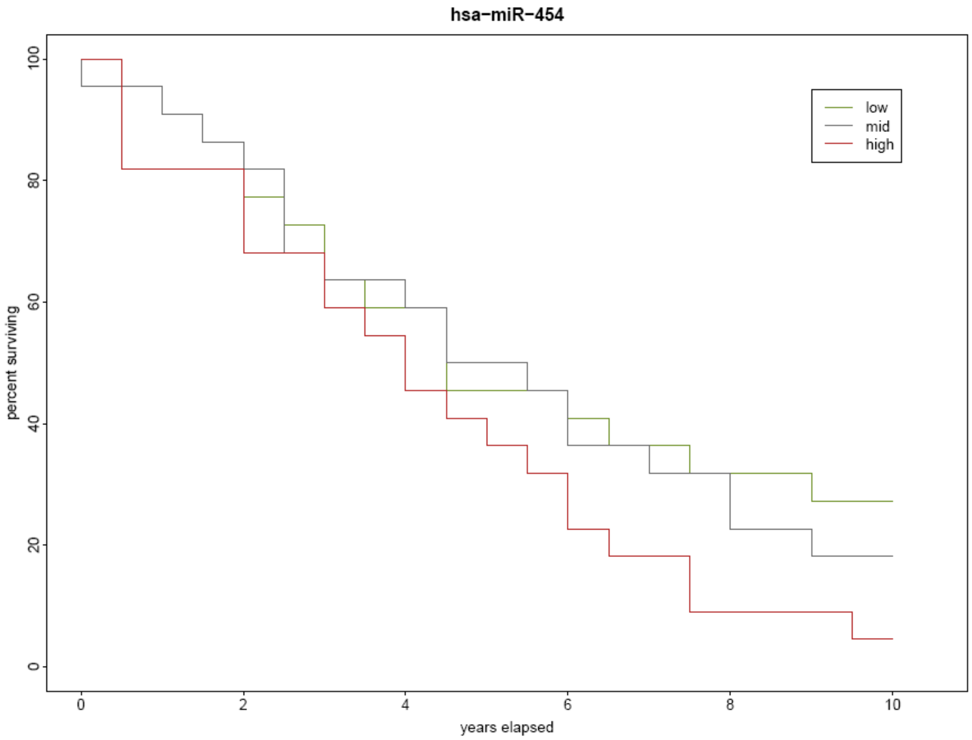

S2 Figure. MicroRNAs and survival. Kaplan-Meier plots of differentially expressed miRNAs using TCGA dataset comprised of a cohort of 65 women with known information of time to death or who lived past 10 years post diagnosis. The cohort was split into three groups of 22, 21, and 22 individuals representing low (green line), middle (blue line), and high (red line) level of respective miRNA.
